# A Provisional Conceptual Framework for Mucosal Colour Assessment in Terrestrial Mammals

**DOI:** 10.3390/ani16111697

**Published:** 2026-06-01

**Authors:** Mette Uldahl, David J. Mellor

**Affiliations:** 1 BrightHorses Institute, Fasanvej 12, 7120 Vejle Øst, Denmark; 2 Animal Welfare Science and Bioethics Centre, School of Veterinary Science, Massey University, Palmerston North 4474, New Zealand; d.j.mellor@massey.ac.nz

**Keywords:** Mucosal colour assessment, veterinary diagnostics, terrestrial mammals, physiological state, colorimetry, Uldahl Standard, tissue perfusion, oxygenation, colour saturation, clinical evaluation, diagnostic standardisation, AI image analysis, spectrophotometry, colour systems, CIELAB, Munsell, ISCC-NBS, animal health and welfare, validation, biomedical imaging

## Abstract

Mucosal colour assessment is widely used in veterinary medicine as an indicator of physiological states, but current approaches are characterized by inconsistent terminology, variable methodology, and differing levels of validation. This communication introduces the provisional Uldahl Standard, a conceptual framework developed to improve consistency, transparency, and reproducibility in mucosal colour assessment in terrestrial mammals. The framework integrates principles from veterinary medicine, colorimetry, and modern imaging technologies, combining perceptual, computational, and instrument-based approaches to colour analysis. Mucosal colour assessment is defined as a multidimensional process comprising colour category, light saturation level, physiological association, assessment method, and level of validation. Nine principal colour categories and eight standardised saturation modifiers were identified through a literature review and incorporated into the framework. The standard further emphasizes transparent reporting of assessment conditions, validation procedures, artefact evaluation, and analytical pathways, including examples using AI-assisted visual analysis. The framework acknowledges the inherent variability in mucosal colour assessment arising from environmental conditions, limitations of human colour perception, and differences in descriptive methods while providing a structured and comparable terminology, linked to defined levels of validation. It is anticipated that application of the proposed Uldahl Standard will provide markedly more robust and consistent descriptions of mucosal colours, which, provided they are combined with well validated clinical signs of the underlying physiology and/or pathophysiology, will greatly enhance the diagnostic power of the procedure.

## Simple Summary

Mucosal colour assessment is commonly used in veterinary medicine to help evaluate physiological and pathological states in animals. However, current approaches are often inconsistent due to differences in terminology, assessment methods, and levels of validation. This communication introduces the provisional Uldahl Standard, a conceptual framework designed to improve consistency, transparency, and reproducibility in mucosal colour assessment in terrestrial mammals. The framework combines principles from veterinary medicine, colorimetry, and modern imaging technologies, integrating visual assessment with computational and instrument-based approaches. The proposed framework defines mucosal colour assessment as a multidimensional process that includes colour category, light saturation level, and human factors in colour assessment methods, and the need for validation of each step in the process. Nine principal colour categories and eight standardized saturation modifiers were identified through a literature review and incorporated into the framework. The standard also emphasizes transparent reporting of assessment conditions, validation procedures, artefact evaluation, and analytical methods, including examples of AI-assisted visual analysis. The framework recognizes that mucosal colour assessment is inherently influenced by environmental conditions, limitations of human colour perception, and differences in descriptive methodology. At the same time, it provides a structured and consistent terminology linked to clearly defined levels of validation.

## 1. Introduction

This communication introduces the Uldahl Standard for mucosal assessment in terrestrial mammals, a provisional conceptual framework for evaluating mucosal colour based on defined colour categories and saturation levels. The model integrates traditional veterinary visual assessment with computationally assisted colour analysis, seeking to enable more systematic evaluation of mucosal colour in relation to physiological state, to support health and welfare assessments.

Assessment of oral mucosae is a long-established component of veterinary clinical examination in domesticated mammals [1]. Compared with skin, mucosal colour is generally easier to evaluate because the lamina propria is thinner than the fibroelastic dermis, allowing underlying vascular and circulatory changes to be more readily visualized at the surface [2,3]. During routine examinations, the mucous membranes of the eye, oral cavity, vagina, and prepuce are readily accessible for assessment [1]. Their colour reflects tissue oxygenation and perfusion [4,5], with normal oxygenated mucosae typically ranging from pink to red depending on species [1].

Mucosal colour assessment is traditionally based on visual evaluation by the clinician, often including descriptors of colour, saturation, and moisture. However, these parameters are inconsistently defined across the veterinary literature. Terminology for normal and abnormal mucosal colour varies considerably between publications and is not always clearly linked to physiological states [6,7,8,9,10,11]. While some sources rely on relative descriptors such as “within normal range” or “paler than normal” [4], human medicine and dentistry have developed colour atlases to support more objective categorization of gingival colour [12,13,14,15,16,17,18].

### 1.1. Colours Commonly Linked to Pathophysiological or Pathological States

Across studies, mucosal colour has consistently been associated with changes in perfusion, oxygenation, inflammation, and tissue viability. Deviations from the normal colour may therefore provide clinically meaningful information about both local pathology and systemic disease [6,7,8,19,20,21]. Critically, because abnormal mucosal colour can arise from multiple causes, findings should always be interpreted in relation to other clinical observations [6,7,19,20,21]. The following consideration of different mucosal colours and inferences of their causes emphasise this point.

Yellow hues are generally associated with altered physiological states such as compromised hepatic function [1]. In clinical medicine, jaundice, characterised by yellowish discoloration of the skin and mucosae, is a recognised sign of liver dysfunction. More specifically, this reflects elevated circulating bilirubin, or impaired bile clearance, with yellow bilirubin deposition visible in the oral mucosa under these conditions. Haemolytic processes can also lead to bilirubinemia [22,23,24]. Additionally, leakage of fibrinogen, necrosis, purulenc, and calcification during inflammatory processes can give rise to yellow tissue [25,26]. Also, chronic gastric dilatation can cause yellow discoloration of mucous membranes [1]. However, yellow colours can also be related to normal physiological appearances, such as in adipose, lymphoid, and sebaceous tissues [26].

Green hues are linked to damage of red blood cells, early necrosis, and may similarly be observed in cholestatic or hepatic conditions. Green coloration appears when haeme is metabolised into the green pigmented chemical biliverdin [27].

Blue hues correspond to cyanosis and hypoxia, reflecting reduced oxygenation or venous pooling [1,2,22,28,29,30]. The development of a generalised blue discoloration can vary from systemic circulatory restrictions, shock (including septic shock), congestion of blood and diminished oxygenation due to varied pathological conditions like heart insufficiency or more local endothelial damage related to viral infection or intoxication [1]. Blue Tongue Virus, as an example, makes blood vessels more permeable, resulting in vasculitis, thrombosis, haemorrhage, oedema, and tissue damage [2,28] within affected tissue [29,30,31].

Purple hues indicate venous congestion and stasis, typically associated with deoxygenated blood or early necrotic changes, producing a purple (violaceus) appearance. In general, conditions associated with a blue discoloration of mucous membranes are comparable to those causing a purple coloration, with purple reflecting a lesser degree of haemoglobin deoxygenation [29,30]. Purpuric conditions, related to thrombocytopenia and vasculitis, can lead to red spots or patches, for example, related to Purpura Haemorrhagica or Equine infectious anemia [22].

In general, pink hues are characteristic of healthy, viable tissue with adequate perfusion and oxygenation [2,32,33]. However, increasing intensity within the pink range, appearing as bright pink or progressing toward red, is associated with hyperaemia, systemic inflammation, and endotoxemia [33].

Red hues are linked to inflammation and reflect hyperaemia and vascular engorgement [1,26]. Red to blue is seen in cyanosis [2,22]. Purpuric conditions, related to thrombocytopenia and vasculitis, can lead to red spots or patches, for example, related to Purpura Haemorrhagica Equine infectious anemia [22].

In mucosal tissue, the appearance of orange hues or intermediate colours often reflects transitional or mixed physiological states, resulting from combined variations in tissue perfusion and blood oxygenation [34].

Brown discoloration is indicative of chronic or advanced pathological processes, associated with pigment accumulation (such as melanin, hemosiderin, or oxidised blood products), reduced or stagnant perfusion, and tissue hypoxia or necrosis. It is frequently observed in conditions involving chronic inflammation, haemorrhage, ischemia, or tissue degeneration [24]. Brown can also be a drug-related discoloration [35] or related to nitrate or nitrite poisoning [1].

A grey coloration is generally indicative of markedly reduced perfusion and oxygenation, reflecting ischemia, shock, or advanced systemic compromise [1]. Diminished capillary flow and low oxygen saturation of haemoglobin result in pale grey to grey-white. mucosal appearances [29,30,31]. As such, grey represents a clinically relevant optical appearance, although it is not a single spectral hue.

It should now be apparent, when interpreting what conditions specific mucosal colours may indicate, why it is critically important to also refer to other clinical observations [6,7,19,20,21].

### 1.2. Colour Saturation or Visual Intensity

In addition to hue (colour), perceived saturation or visual intensity and vividness of mucosal colour, reflect the combined effects of chroma (the colour intensity) and lightness (how light or dark the colour is). These features play a critical role in clinical interpretation as they primarily reflect the concentration and oxygenation state of haemoglobin within superficial capillary beds [30]. They are thereby influenced by tissue perfusion, vascular volume, and oxygen delivery [29].

In healthy tissue, colour saturation or visual intensity is typically low to moderate, observable as a well-perfused pink, corresponding to balanced perfusion and oxygenation [29].

Markedly reduced saturation may indicate hypoperfusion, anaemia, or degenerative processes [1,22]. Increasing saturation (moderate to strong or vivid) is often associated with hyperaemia, inflammation, shock, or vascular engorgement [31]. Dark or deep tones frequently reflect venous congestion, reduced oxygenation, diseases of the heart or brain, fever, or tissue stasis, and they may precede cyanotic or necrotic changes depending on hue [1,22].

### 1.3. Predicting or Diagnosing Health and Welfare Risks

Establishing relationships between mucosal colour (including saturation) and physiological state are key requirements for predicting or diagnosing health and welfare risks. Accordingly, optimal colour assessment is essential to enable reliable differentiation between clinically normal and abnormal conditions. At the same time, linkages between assessed colours and specific local or systemic functional states must be reliable. This would require indices that can detect related circulatory and tissue functions, including perfusion, microcirculatory dynamics, vascular congestion (e.g., venous pooling), oxygenation, capillary refill, vasodilatation, and haemoglobin concentration. In addition, factors such as tissue integrity, tissue hydration, metabolic and toxicological influences, pigment accumulation, and the deposition of cellular degradation products (e.g., bilirubin) contribute to observed colour variations.

### 1.4. Colours Plus Tissue Integrity May Indicate Pain

Potential associations with pain are also relevant, particularly as behavioural and physiological responses may coincide with changes in mucosal appearance. Pain may arise from partial or complete obstruction of venous and lymphatic outflow, leading initially to venous congestion, oedema, and accumulation of deoxygenated blood distal to the constriction [3,29]. If constriction persists, arterial inflow may become compromised, causing ischemia, tissue injury, and eventually necrosis [29].

Restricted blood flow contributes to pain through oedema-related tissue distension, ischemia-induced activation of nociceptors, and direct nerve compression or injury [36,37,38,39]. In advanced stages, pain may diminish due to ischemic nerve damage, which is not clinically reassuring as it may indicate worsening tissue injury [40].

Colour changes reflect these underlying hemodynamic alterations. Venous obstruction typically causes progression from red to purple or blue discoloration [3,39] while advancing arterial compromise may lead to grey (mottling) and eventually black necrotic tissue [41,42]. Venous congestion itself is associated with pain, with erythema (abnormal; redness of the skin or mucosae) and purple to dusky-purple discoloration linked to increasing pain severity [39,41,43,44].

### 1.5. Requirements for Consistency in Mucosal Colour Assessment

To this point, it may appear that the case has been made to abandon the objective of more consistently and accurately utilising mucosal colour as a diagnostic tool. However, the overall purpose has been to highlight key issues that hinder reliability to facilitate the next step in the process, which is to assess what can be achieved despite continuing constraints.

Because mucosal colour assessment is often used as an indicator of mammalian health and welfare, the development of a validated and standardized framework for interpretation is essential. Studies in veterinary education have shown that students assess mucosal colour equally well using either colour charts or word-based scales. However, both methods are associated with consistent variability and some degree of assessment error [45], highlighting the need for a standardized system that enables reliable evaluation of assessment methods and identification of which approaches perform best at different levels of clinical training or expertise.

A major challenge in achieving consistency lies in defining unambiguous colour terminology and linking descriptive assessments to objective colour values within a standardized system. Modern vision science and colorimetry demonstrate that human colour perception exists within a multidimensional non-linear space [46,47], influenced not only by optical properties but also by psychophysical, physiological, and psychological variables [35,46,48,49]. This complexity contributes to variability in the interpretation and reporting of mucosal colours across current veterinary literature and practice.

The lack of validation, standardisation, and consistent terminology complicates clinical communication, limits comparability between studies, and may impair training in reliable colour assessment. Developing a standard for mucosal colour assessment in terrestrial mammals therefore requires an integrative approach that bridges perceptual, conceptual, and instrument-based colour models. Central to this is the alignment of standardized terminology with internationally recognized colour systems and colorimetric measurements to reduce subjectivity while maintaining clinical applicability.

The provisional conceptual framework presented here acknowledges the inherent variability in mucosal colour assessment arising from environmental conditions, limitations of human colour perception, and differences in methods used for colour description. However, it provides a structured and transparent terminology that clarifies how assessments have been performed and the corresponding level of validation.

Within the provisional Uldahl Standard, mucosal colour assessment is defined as a multidimensional evaluation comprising:

- defined colour categories and saturation levels
- standardised assignment of colour names
- use of internationally recognised colour systems
- optional methods for mucosal colour assessment
- defined levels of validation.

## 2. Proposal for a Provisional Conceptual Framework for Assessment of Mucosal Colour in Mammals

Human colour perception is a highly complex process influenced by multiple physiological, psychological, and environmental factors. As a result, colour assessment faces significant challenges in achieving objectivity while remaining practical and clinically meaningful. Nevertheless, it is possible to establish validated levels of assessment in which the degree of objectivity and reliability is clearly defined, and in which associations with physiological states, if definable, can be evaluated for significance.

The development of a validated colour standard would improve consistency, reliability, and comparability across clinical practice, education, and research. Establishing such a framework requires clearly defined colour categories linked to objective colour values within a recognised metric colour system.

The Uldahl Standard is based on the internationally recognized ISCC-NBS colour naming system, selected for its practical terminology and compatibility with established colorimetric systems [50,51,52]. The system is grounded in the core dimensions of colorimetry: *hue* (the color family), *chroma* (color purity), and *lightness* (perceived brightness), which together underpin colour classification and naming. Colours may further be refined into primary and secondary components (e.g., pinkish brown) [50,51,52,53,54].

An additional measure, “saturation,” is used within the Uldahl Standard to refine colour descriptions further (see Table 1). Saturation is a perceptual attribute related to colour purity (chroma) and perceived brightness (lightness) and is described using formal qualitative modifiers derived from the literature [55,56]. These descriptors include pale, light, moderate, strong, vivid, deep, dark, and very dark, representing increasing intensity [51,55,56,57].

Composite or informal descriptors, such as bright, muddy, toxic, or dull, are excluded because they combine multiple perceptual dimensions and are not reliably reproducible. Likewise, surface descriptors such as moist, dry, glossy, or shiny are excluded, as they describe optical surface properties, rather than colours themselves [58].

Based on the literature, nine mucosal colour categories relevant to assessment were identified as primary names: yellow, green, blue, purple, pink, red, orange, brown, and grey. These are arranged along a colour scale aligned with the ISCC-NBS system, as are corresponding secondary colour names [55] (see Table 2).

A third parameter, physiological association, links colour and saturation to underlying physiological or pathological states (see Table 3). This reflects the principle that mucosal colour assessment gains clinical relevance only when interpreted in relation to health and welfare. Future validation studies may establish predictive associations between specific colour-saturation combinations and physiological conditions.

Accordingly, the Uldahl Standard comprises three integrated parameters: colour, saturation level, and physiological association (see Table 3).

## 3. Application of the Uldahl Standard

Mucosal colour assessment at a validated level requires a systematic reporting and evaluation system of data quality, supported by clearly defined protocols for both in vivo and photographic material. To ensure transparency, reproducibility, and comparability across studies, it is essential that key parameters are explicitly defined, evaluated, and reported [50,53,54,58,59]. These should include, among others, the type of species and mucosa, the conditions during the assessment, validation of source material, documentation of method for assigning colour names, identification and management of artefacts, and validation of physiological state. See Table 4.

### 3.1. Type of Mucosa and Species

**Figure 1:**
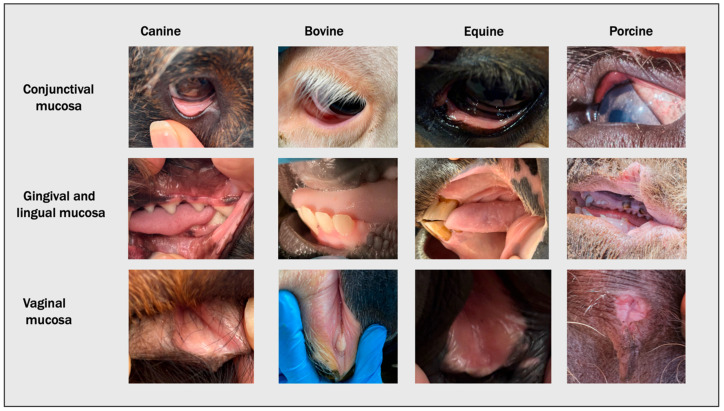
Different types of mucosal surfaces: conjunctival, gingival, lingual, and vaginal in four mammal species; canine, bovine, equine, and porcine.

The type of mucosa and species evaluated should be reported. See Figure 1 and Table 4.

### 3.2. Validation of Physiological State

The physiological state of the animals should be verified at a clearly defined level, such as through owner reported information or veterinary assessment [60,61]. Where relevant, it should also be specified whether associations with pain were evaluated, and if so, which methods were used for the assessment, and the features of the pain detected (see Table 4).

Mucosal colour and perceived saturation can change rapidly in response to alterations in circulatory state, including events such as death. It is therefore essential to assess and document mucosal colour in close temporal relation to the relevant event, as changes may result in altered or blanched appearance; for example, post-mortem at autopsy [22].

### 3.3. Assessment Conditions

Environmental conditions during assessment, including the use of natural or artificial light, should be reported. Use of natural light is preferred [1,55] (see Table 4).

Ideally, lighting conditions should be standardised or thoroughly documented to minimise variability in colour perception. However, as this is not always feasible, robust validation of the source material may help compensate for such limitations (see Section 3.4).

### 3.4. Validation of Source Material

Mucosal surfaces may be assessed either in vivo or by photographic material. Validation of colours can occur at multiple levels [62]. Direct visual assessment and description by a veterinarian is considered to represent a high level of validation [60]. Other levels may include assessment by trained professionals or owner-reported observations [61].

When photographic material is used for subsequent colour analysis, mucosal colour should be validated in vivo and subsequently compared directly with the photographic representation. These types of data require confirmation that the colours observed in the image are consistent with those assessed in vivo (see Table 4).

### 3.5. Artefact Assessment

**Figure 2:**
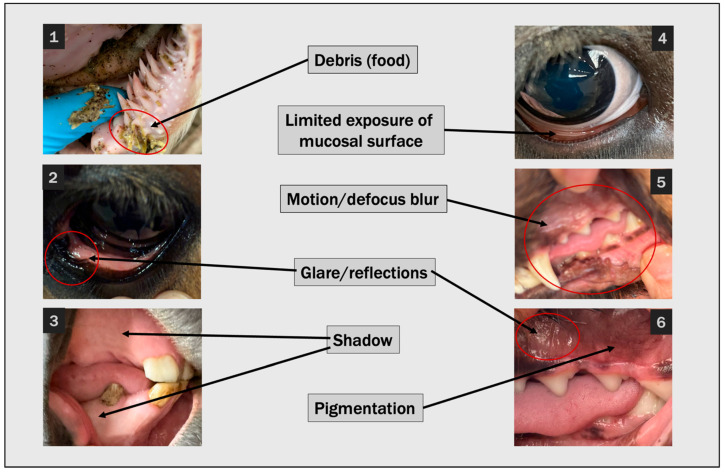
Illustration of different types of artefacts which interfere with mucosal colour assessment. (1) debris from food covering bovine oral mucosa; (2) Glare/reflection on equine conjunctival mucosa; (3) shadow on a part of the equine oral mucosa; (4) limited/insufficient exposure of mucosae of bovine conjunctival mucosa; (5) motion blur/defocus on image of canina oral mucosa; (6) Glare/reflections and pigmentation on canine oral mucosa.

For both in vivo and photographic analyses, the approach to artefacts evaluation should be clearly reported, including the types of artefacts considered (e.g., shadows, motion or defocus blur, glare, pigmentation, or debris covering mucosal surfaces) [58,63]. These artefacts are illustrated in Figure 2.

In addition, clearly defined criteria for the inclusion and exclusion of observations and images should be established and documented (see Table 4).

### 3.6. Documentation of Colour System and Assessment Method Used for Assigning Colour Names

The system used for describing colours should be reported. Internationally recognized colour description systems should be used to ensure consistent and reproducible colour descriptions [50,51,53,54,55,57].

**Figure 3:**
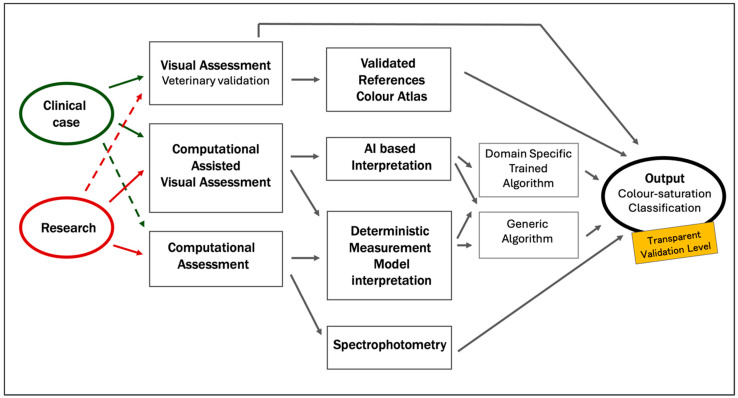
The diagram illustrates different methods for assessing mucosal colour, all of which can be applied within the Uldahl Standard. However, the level of validation varies between methods, which makes some approaches more suitable for research purposes than others (dotted arrows indicate lower suitability).

The method used for assigning colour names should be clearly documented. Observer-dependent methods (visual assessment with or without a reference to a validated standard or colour chart), reduced observer dependence (computational analysis using AI algorithms with or without visual co-evaluation of a trained professional), and observer independent methods (spectrophotometry or computational deterministic colorimetric models) can all be used in applications of the Uldahl Standard [59,62,64,65] (see Figure 3). However, the level of validation of different methods output varies considerably, which is why declaration and transparency regarding methodology in colour analysis is essential [45,59,62] (see Table 4).

## 4. Examples of Application of the Uldahl Standard

To illustrate how the Uldahl Standard can be applied in practice across research, education, and clinical purposes, three examples are provided below. The assessment method used in these examples is an AI-assisted visual colour analysis. However, the Uldahl Standard is not limited to this approach. Any type of analysis, including purely visual assessments, as well as fully deterministic computational analyses, can be incorporated within the standard. The only requirement is that the analytical method is clearly reporting as part of the validation process (see Section 3.6 and Table 4).

Samples from three sets of photographic material (Examples 1, 2, and 3) were analysed descriptively using a large language model (ChatGPT, GPT-5.2, OpenAI, San Francisco, CA, USA). Standardised colour terminology was assigned to each sample using the prompt “Please provide the Munsell code and ISCC-NBS name for the colour in the attached sample”. For each sample, a Munsell notation describing hue, value, and chroma was generated [51,53,54], along with a corresponding colour name based on the ISCC-NBS system.

The ISCC-NBS designation provided a classification of colour (hue) in combination with modifiers reflecting saturation (i.e., chroma and/or lightness). Each sample was thereby assigned a colour category and a corresponding formal qualitative descriptor for saturation level. Where applicable, both primary and secondary colour components were identified; in some cases, only a primary colour was assigned.

Following the initial AI-based categorisation, a visual quality control was performed by a trained professional (veterinarian; one of the authors: MU) to assess consistency of the assigned colour with the observed sample.

Provided below are three specifically different examples. Example 1 presents a general report, Example 2 illustrates how mucosal colour varies due to anatomical and physiological differences across tissue sites, and Example 3 demonstrates the impact of artefacts on analysis results.

### 4.1. Example 1: Veterinary Validation Without a Validated Reference to Physiological Interpretation

**Figure 4:**
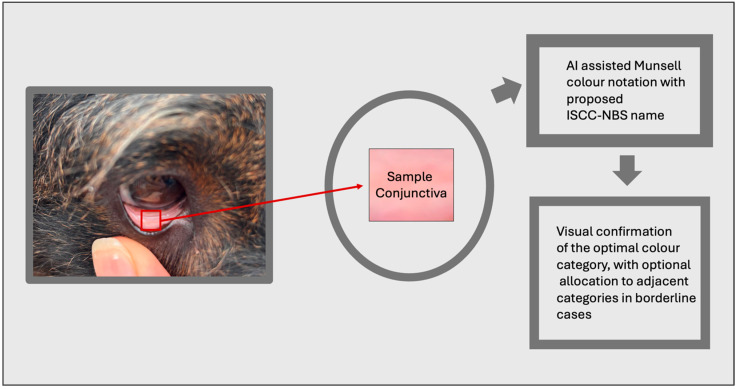
Sample: canine conjunctival mucosa. A combined AI assisted visual assessment method was used for analysis of samples.

A conjunctival mucosal sample (lower lid) was obtained from a clinically normal canine (breed Dachshund; age 3 years; sex: female) (see Figure 4). The physiological status of the animal was confirmed through veterinary assessment as clinically normal. Colour analysis was performed using photographic material captured under natural daylight conditions. A minor level of glare/reflection was present, leaving a substantial portion of the exposed mucosa to be of sufficient quality to allow reliable interpretation.

The results were: Munsell notation: 5R 7/4; ISCC-NBS name: ‘light pink’. The colour classification obtained through AI based analysis was independently confirmed by visual assessment conducted by a trained professional (veterinarian). The classification of ‘light pink’ was consistent with a physiologically normal state. This finding aligned with expected mucosal colour in a healthy canine, reflecting normal perfusion and oxygenation.

### 4.2. Example 2: Veterinary Validation Without a Validated Reference to Physiological Interpretation: Mucosal Colour Variation Between Anatomical Sites

**Figure 5:**
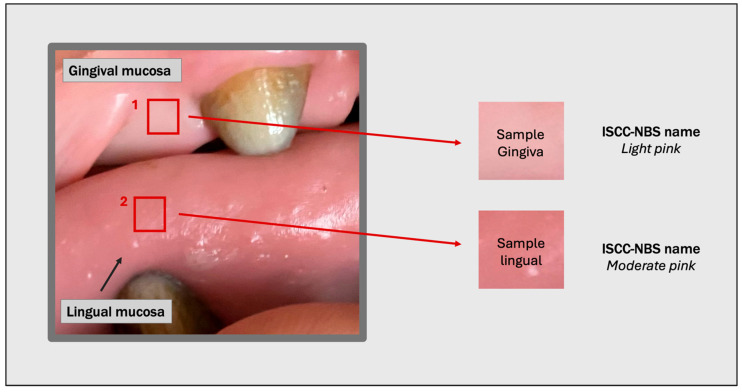
Sample: Equine oral mucosa. A combined AI assisted visual assessment method was used for analysis of samples. The example demonstrated variation of mucosal colour between anatomical sites.

Two samples were obtained from a horse (breed, sports pony; age 8 years; sex: gelding), representing (1) the gingival mucosa and (2) the lingual mucosa. (see Figure 5). The physiological status of the animal was confirmed through veterinary assessment as clinically normal.

In vivo veterinary assessment of mucosal colour was conducted, followed by direct comparison with the photographic representation. Consistency between in vivo observations and the photographic images was confirmed. Colour analysis was performed using an AI-based approach grounded in the Munsell colour system, with subsequent conversion to a standardised colour name using the ISSCC-NBS colour naming system.

The results for Sample 1 (Gingival mucosa): Munsell notation: 5R 7/3; ISCC-NBS name: ‘light pink’. Sample 2 (Lingual mucosa): Munsell notation: 5R 6/4; ISCC-NBS name: ‘moderate pink’. The colour classification obtained through AI-based analysis was independently confirmed by visual assessment conducted by a trained professional (veterinarian). It demonstrated colour variation between the two anatomical sites, with the lingual mucosa exhibiting higher saturation (‘moderate pink’) compared to the gingival mucosa (‘light pink’). Both findings are consistent with a physiologically normal state. This pattern aligned with expected mucosal colour variation between anatomical sites in healthy equines, reflecting normal perfusion and oxygenation.

### 4.3. Example 3: Owner-Reported Images Without a Validated Reference to Physiological Interpretation: The Effect of Lighting Artefact

**Figure 6:**
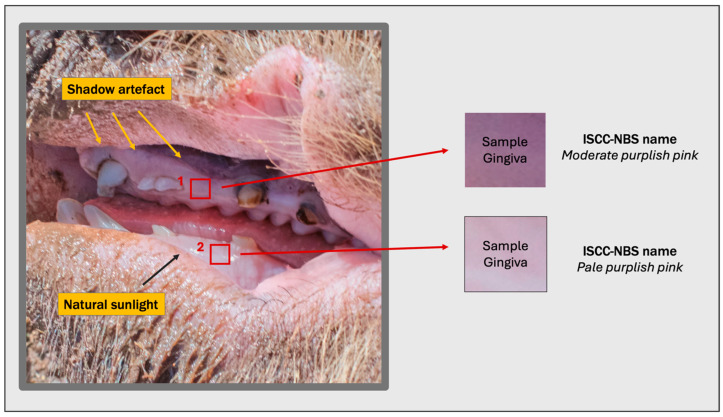
Sample: porcine gingival mucosa. A combined AI assisted visual assessment method was used for analysis of two samples. The effect of lightning artefact is demonstrated.

Two lingual mucosal samples were obtained from a porcine (breed Mangalica; age 1 year; sex: female), representing: (1) the maxillary region and (2) the mandibular region (see Figure 6). The physiological status of the animal was not confirmed through veterinary assessment but was owner-reported as healthy. The photographic material was owner-reported and captured under natural, sunny, daylight conditions. The maxillary region was affected as shadowed, whereas the mandibular region was exposed to direct sunlight, resulting in uneven illumination and the presence of artefacts.

No in vivo veterinary assessment of mucosal colour was conducted, and consistency between in vivo observations and the photographic images was not verified. Consequently, the assessment was based solely on the screen-dependent visual interpretation.

Colour analysis was performed using an AI-based approach grounded in the Munsell colour system, with subsequent conversion to a standardised colour name using the ISSCC-NBS colour naming system. The results for Sample 1 (maxillary region) were Munsell notation: 5RP 7/4; ISCC-NBS name, ‘Moderate purplish pink’. For sample 2 (mandibular region), there were Munsell notation: 5RP 8/2 and the ISCC-NBS name: ‘Pale purplish pink’. The colour classifications obtained through AI-based analysis were independently confirmed by visual assessment conducted by a trained professional (veterinarian).

Due to the combined absence of in vivo validation and lack of veterinary confirmation of physiological status, the results cannot be reliably related to the physiological state of the animal. Furthermore, the presence of lighting artefacts, especially shadow in the maxillary region and direct sunlight exposure in the mandibular region, influenced the perceived colour and saturation. The shadow artefact in the maxillary region likely contributed to an apparent increase in saturation relative to the mandibular region.

Given the lack of validation and the presence of significant artefacts, the photographic material was not suitable for drawing veterinary conclusions regarding the health or welfare status of the animal.

## 5. Concluding Comments

Visual evaluation of oral mucosal surfaces in terrestrial mammals is a recognised and integral component of clinical examinations in veterinary medicine. It has long been applied to assess local and systemic health and welfare conditions and remains an important part of routine clinical practice. Despite its widespread application, a scientifically validated and standardised framework for mucosal colour assessment has not previously been established.

Human colour perception is influenced by multiple factors, making objective colour assessment inherently challenging while maintaining clinical practicality and relevance. Nevertheless, validated levels of assessment can be established in which objectivity and reliability are clearly defined, allowing systematic evaluation of associations between colour characteristics and physiological states.

Although oral mucosal colour has been incorporated into numerous published studies, the terminology and descriptors applied have varied across clinical contexts and methodologies. These observations highlighted the need for a more structured and scientifically grounded assessment framework for use in clinical practice, education, and research.

The Uldahl Standard was developed as a provisional conceptual framework for the evaluation of mucosal colour. The framework was informed by a review of existing literature, systematic assessment methods, and internationally recognised colorimetric systems. From this process, two principal analytical dimensions were defined; colour and saturation level, treated as separate but complementary components of assessment.

The framework recognizes that mucosal colour assessment is inherently variable due to environmental influences, limitations of human colour perception, and differences in prescriptive methodology. Nevertheless, it establishes a structured and comparable terminology that improves transparency by defining how assessments are performed and the level of validation.

Within the Uldahl Standard, nine colours considered relevant to mucosal assessment were identified: green, yellow, blue, purple, pink, red, orange, brown, and grey. In addition, eight saturation levels were defined as formal quantitative descriptors: pale, light, moderate, strong, vivid, deep, dark, and very dark. The descriptors reflect the way colour is commonly assessed in clinical practice, where evaluation involves both qualitative perception and semi-quantitative assessment of colour intensity.

Application of the Uldahl Standard enables structured classification of mucosal colours through primary and secondary colour categories in relation to potential diagnostic relevance. The framework accommodates assessment approaches, ranging from subjective visual evaluation to objective instrument-based or computational analyses, thereby supporting both clinical practice and research. Examples of applications using computationally assisted visual assessment have been presented.

The provisional conceptual framework should be regarded as an evolving framework, intended to support a more systematic understanding of the clinical implications of oral mucosal appearance.

It is anticipated that application of the proposed Uldahl Standard will provide markedly more robust and consistent descriptions of mucosal colours which, when combined with well validated clinical signs of the underlying physiology and/or pathophysiology, when available, will greatly enhance the diagnostic power of the procedure.

## Figures and Tables

**Table 1:** Overview ISCC-NBS saturation descriptors included in the Uldahl Standard and their dominant relation to either lightness or chroma.

ISCC-NBS Saturation Descriptors Included in the Uldahl Standard	The Descriptors Dominant Colour Property
Pale/very light	Lightness
Light	Lightness
Moderate	Lightness
Strong	Chroma
Vivid	Chroma
Deep	Chroma
Dark	Lightness
Very Dark	Lightness

**Table 2:** Overview of colours included in the Uldahl Standard with their corresponding primary and secondary colour names.

ISCC-NBS Colour Name for Colours Included in the Uldahl Standard	
Primary Colour Names	Secondary Colour Names
Yellow	Yellowish
Green	Greenish
Blue	Blueish
Purple	Purplish
Pink	Pinkish
Reds	Reddish
Orange	Orangish
Brown	Brownish
Grey	Greyish

**Table 3:** Overview of the Uldahl Standard for mucosal colour assessment, comprising three integrated parameters: colour, saturation level, and physiological association.

Parameter	Description	Example
Colour	Defined within an internationally recognised colour system and aligned with the corresponding ISCC-NBS colour names	Munsell notation: 5YR 5/4 ISCC-NBS name: moderate brownish pink
Saturation descriptors	ISCC-NBS modifier describing perceived intensity through variation in lightness or chroma	Lightness modifiers: *pale*, *dark* Chroma modifiers: vivid, strong
Physiological Association	Association between colour-saturation combinations and physiological states	Light pink: observed in clinically normal mammals

**Table 4:** Components and acceptance criteria of the Uldahl Standard for mucosal colour assessment.

Component	Specification	Required	Notes
Mucosal types	All relevant mucosal surfaces	All accepted	Specify species and anatomical site(s) See Section 3.1
Physiological state	Normal vs. abnormal	Yes	Pathology clarified if applicable See Section 2 and Section 3.2
Environmental conditions	All relevant parameters	Conditional acceptance	Parameters such as lighting conditions (daylight/artificial light) See Section 3.3
In vivo validation	Veterinary/other	Yes	Must be disclosed See Section 3.4
Image validation	Photo image and in vivo well matched	Yes, if use of images	Veterinary/trained professional preferred See Section 3.4
Artefact screening	Shadow, glare, debris, etc.	Yes	Criteria must be defined for inclusion/exclusion See Section 3.5
Assessment method	Visual/AI/instrument-based	All accepted	Assessment method must be reported See Section 3.6
Assignment of colour name (hue)	9 categories (Yellow-grey)	Yes	Use Uldahl Standard colours See Section 2 and Section 3.6
Assignment of saturation descriptor	8 modifiers (Pale to very dark)	Yes	Use Uldahl Standard saturation descriptors See Section 2 and Section 3.6

## Data Availability

No new data were created or analyzed in this study.

## Abbreviations

The following abbreviations are used in this manuscript:

AI	Artificial Intelligence
CIELAB	Commission Internationale de l’Éclairage *
ChatGTP	Chat Generative Pre-trained Transformer
ISCC	Inter-Society Colour Council
L*A*B*	Three-dimensional colour space (L* = lightness; A* = Green-red axis; B* = Blue-yellow axis)
NBS	U.S. National Bureau of Standards
